# Systemic inflammatory syndromes as life-threatening side effects of immune checkpoint inhibitors: case report and systematic review of the literature

**DOI:** 10.1136/jitc-2022-005841

**Published:** 2023-03-06

**Authors:** Lisa L Liu, Marcus Skribek, Ulrika Harmenberg, Marco Gerling

**Affiliations:** 1 Department of Oncology-Pathology, Karolinska Institute, Stockholm, Sweden; 2 Theme Cancer, Karolinska University Hospital, Stockholm, Sweden; 3 Department of Biosciences and Nutrition, Karolinska Institute, Stockholm, Sweden

**Keywords:** Melanoma, Lung Neoplasms, Inflammation Mediators, Immunotherapy

## Abstract

Immune checkpoint inhibitors (ICIs) are associated with a wide range of immune-related adverse events. As oncological indications for ICIs widen, their rare side effects become increasingly visible in clinical practice and impact therapy decisions.

Here, we report a rare case of early-onset, mild cytokine release syndrome (CRS) in a patient who received ICIs for a metastasized renal cell carcinoma, which led to treatment discontinuation.

We further provide a systematic review of the literature of CRS and related life-threatening side effects of ICI treatment, such as hemophagocytic lymphohistiocytosis (HLH). We searched Medline, Embase and the Web of Science Core Collection from inception to October 2021 for reports on CRS, cytokine storm, macrophage activation syndrome, HLH, and related hyperinflammatory disorders in patients with solid cancers receiving ICIs. We found n=1866 articles, which were assessed for eligibility independently by two examiners. Of those, n=49 articles reporting on n=189 individuals were eligible for review. We found that the median time from last infusion to the occurrence of CRS/HLH was approximately nine days, while the onset of symptoms varied from immediately after infusion to one month after treatment. Most patients were treated with either corticosteroids or the anti-interleukin 6 (IL-6) antibody tocilizumab, and although the majority of patients recovered, a few cases were fatal. Concomitant IL-6 and ICI treatment were reported as beneficial for both the antitumoral effect and for limiting side effects. Data from international pharmacovigilance databases underscored that ICI-related CRS and HLH are rare events, but we identified significant differences in reported frequencies, which might suggest substantial under-reporting.

The results from this first systematic review of CRS/HLH due to ICI therapy highlight that life-threatening systemic inflammatory complications of ICIs are rare and might be associated with fatal outcome in approximately 10% of patients. Limited data support the use of IL-6 inhibitors in combination with ICIs to augment the antitumoral effect and reduce hyperinflammation.

## Introduction

Immune checkpoint inhibitors (ICIs) have become important therapeutic options for various tumor types. ICIs are associated with specific toxicities, commonly referred to as immune-related adverse events (irAEs), which can lead to treatment interruption or discontinuation. International guidelines aid clinicians in the diagnosis and management of relatively common irAEs, such as skin rashes, colitis, thyroiditis, and pneumonitis.^1 2^ However, the increasing volume of patients treated with ICIs is starting to reveal less common side effects, including systemic hyperinflammatory syndromes. Nonspecific systemic inflammatory reactions to ICIs, such as self-limiting fever or skin rashes during or shortly after infusion^2^ need to be distinguished from severe, persistent, and potentially life-threatening conditions such as cytokine release syndrome (CRS) and hemophagocytic lymphohistiocytosis (HLH)/macrophage-activation syndrome (MAS).^3^ Due to their severity, these irAEs are of particular clinical importance and require a decision on continuation of ICI treatment, for which evidence is lacking. Because of their rarity, hyperinflammatory syndromes are incompletely captured in randomized clinical trials with ICIs^4 5^ and most international irAE guidelines lack specific recommendations for their management.^1 2^ Although a recent irAE guideline from the Society of Immunotherapy for Cancer discusses HLH as an irAE with potentially high lethality, no specific treatment recommendation could be made.^6^ Hence, real-world data and case reports of rare irAEs are needed to understand their frequency and severity, and to improve clinical management.

In cancer therapy, CRS is best understood in the context of chimeric antigen receptor (CAR) T cell therapies, where it occurs in a substantial proportion of patients at different levels of severity.^7^ CRS is believed to be mainly driven by T cell-derived interferon gamma (IFN-γ), which stimulates macrophages to produce various proinflammatory substances including interleukin 6 (IL-6) and tumor necrosis factor alpha (TNF-α).^8^ Therapeutically, IL-6 inhibition with specific anti-IL-6 receptor antibodies, such as tocilizumab, has proven highly effective against CRS, reflected by the US Federal Drug Agency’s approval of tocilizumab for CAR T cell-induced CRS.^9^ This is important because IL-6 inhibition could allow for the continuation of any treatment associated with mild CRS.^10^

HLH is an umbrella term for life-threatening hyperinflammatory conditions with supramaximal activation of the immune system. For the diagnosis of HLH, the HLH-2004 diagnostic criteria are frequently used, although they were developed for the pediatric population.^3^ The criteria include clinical features such as fever and splenomegaly, as well as laboratory findings such as cytopenias, hypertriglyceridemia, hyperferritinemia, evidence of hemophagocytosis, and the absence of natural killer (NK) cell activity.^3^ More recently, the HScore, which includes similar criteria to HLH-2004, was developed to estimate the probability for reactive HLH in adults with inflammatory syndromes.^11^ Genetic analyses from pediatric patients have revealed a wide variety of predisposing variants that presumably play a role for different immune cell types, suggesting that HLH-related conditions represent a complex disease continuum.^12^

Reports on hyperinflammatory syndromes due to ICI treatment have started to emerge during recent years, and have suggested that these are relatively rare, but potentially life-threatening events.^13^ Both HLH and CRS can be fatal by causing hypotension, capillary leak syndrome, and consequently organ dysfunction.^8^ Because of the potential risk of increasing the severity of CRS/HLH on repeated exposure to a particular trigger, suspicion of these hyperinflammatory syndromes in clinical practice most often leads to treatment cessation. Hence, a better understanding of this complex disease spectrum in the context of ICI treatment is needed to guide decision-making on treatment continuation and optimal management.

Here, we present a rare case of early-onset CRS after ICI treatment of a metastatic renal cell carcinoma. To set the stage for a more evidence-based approach to CRS, we provide a systematic review of the literature, in which we identify n=49 articles on n=189 patients with hyperinflammatory syndromes due to ICIs. The results reveal that most patients with CRS/HLH recovered and that a fatal outcome occurred in approximately 10% of all patients. In addition, the literature reveals that corticosteroids and IL-6 inhibition may provide effective therapies. Extrapolating from preclinical data, which we review in brief, we posit that rechallenging with ICIs after CRS/HLH should be considered at least in patients with mild hyperinflammatory ICI side effects who are expected to benefit from ICI treatment. Recent data suggest that ICIs in combination with IL-6 antagonists may boost the antitumoral effect, while simultaneously protecting from severe irAEs, which makes ICIs in combination with IL-6 inhibition an attractive option for rechallenge that warrants further research.

## Methods

### Case report

The clinical case of CRS was encountered by the authors in their clinical practice at Karolinska University Hospital, Stockholm, Sweden. Clinical data were gathered through review of the electronic patient journal. Data were visualized using GraphPad Prism V.9.4.0.

### Systematic review

We performed a systematic search for published reports on CRS, HLH and related hyperinflammatory diseases in MEDLINE, Embase, and the Web of Science databases as of October 2021. The search strategies were designed in collaboration with the Karolinska Institute library and are included as online supplemental figure 1. Deduplication was performed as previously described.^14^

10.1136/jitc-2022-005841.supp1 Supplementary data

Additional manual searches were performed based on the lists of references in the eligible studies, and a reduced set of keywords in the MEDLINE database only, as of June 13, 2022. Inclusion criteria for abstract review were assessed independently by two reviewers (LLL and MG) and defined as follows: (1) report on any hyperinflammatory syndrome (CRS, HLH, MAS, or any indication of these or related systemic syndromes) on human patients with solid tumors, (2) reported use of any ICI at any time of the treatment, (3) case series or case reports, that is, no randomized controlled trial, unless explicitly stated that CRS/HLH/MAS or any other hyperinflammatory syndrome was reported, (4) articles in English, Swedish, Chinese or German. LLL and MG agreed on article inclusion by discussing the articles for which the initial decision on inclusion differed. For all eligible articles, the full-text was downloaded by LLL or MG. Data were extracted by either LLL, MS, or MG, and all authors convened to agree on final inclusion. As the majority of the included studies were case reports on rare events, no further criteria for study quality assessment were applied and the risk for bias was not assessed. The systematic review was not preregistered.

## Results

### Case report

A patient in her early 70s had presented to her primary care physician with increasing fatigue and right upper quadrant pain radiating to the spine. She reported involuntarily weight loss of 20 kg during the last year. She had a history of arterial hypertension, hyperlipidemia, and she had been a smoker for 35 years, but had quit more than 10 years previously. No history of allergic reactions was recorded. At the time of presentation, she was on treatment with metoprolol, amlodipine, enalapril, atorvastatin, and ketoprofen. Workup with a CT scan revealed a 12.5×8×15 cm multicystic renal tumor, lymph node metastases, at least two liver metastases, the largest of which was 5 cm in diameter, three pulmonary/pleural metastases, and a bone metastasis in the left acetabulum. A needle biopsy from the lesion in the right kidney showed clear cell renal cell carcinoma, grade 2 according to the International Society of Urologic Pathologists. She had a performance score of 2 according to the Eastern Cooperative Oncology Group (ECOG). According to the International metastatic RCC database consortium prognostic criteria,^15^ she had a poor prognosis due to anemia, elevated neutrophils and a short period from diagnosis to start of systemic treatment. For the pain, the patient was started on paracetamol and oxycodone, which she used sparingly, and she needed transfusions because of low hemoglobin levels.

**Figure 1:**
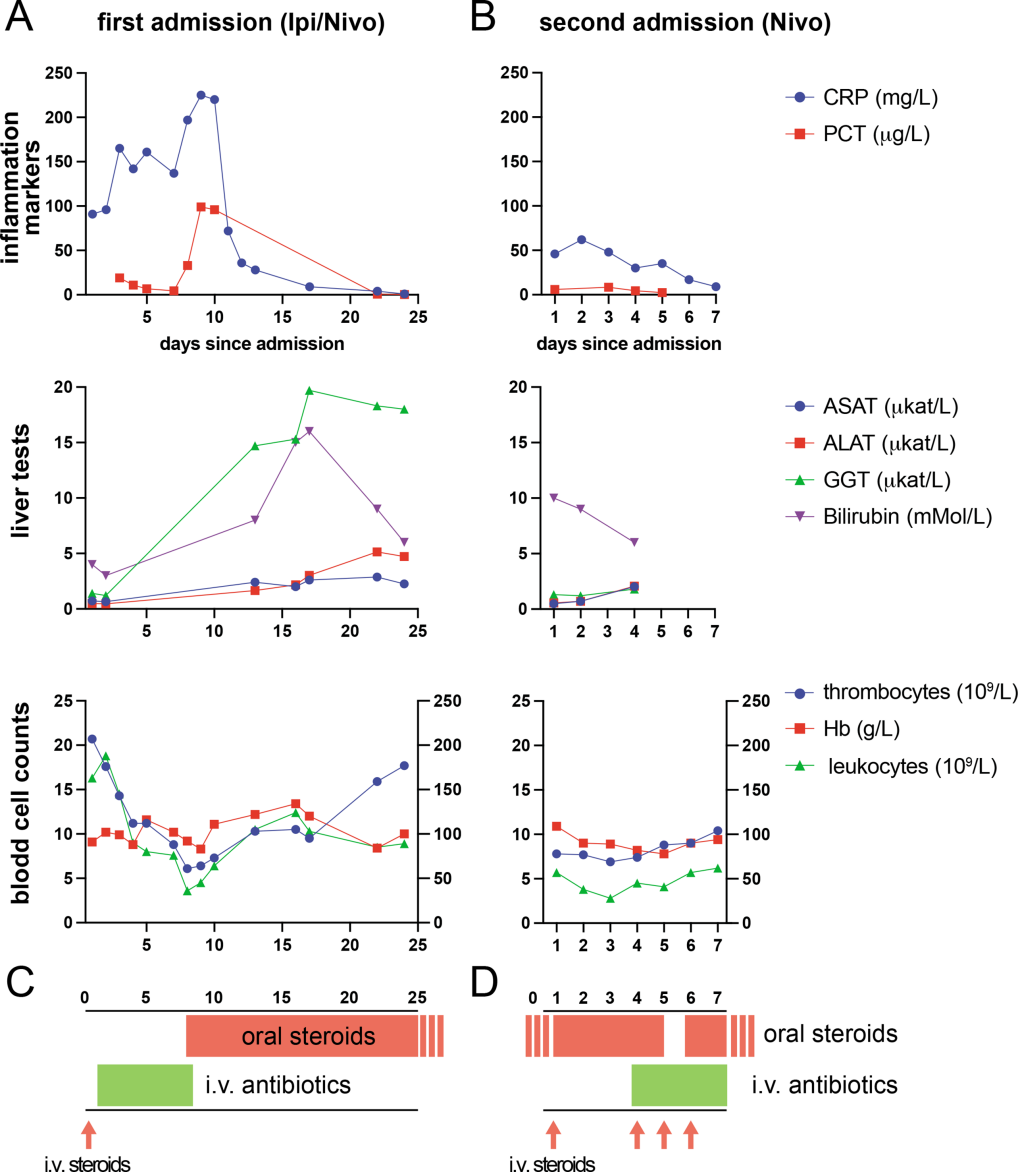
Laboratory tests after immune checkpoint inhibitor (ICI) treatment in a patient with metastatic renal cell carcinoma. (A) Routine blood tests for the indicated markers at the first admission after treatment with ipilimumab and nivolumab; legend as indicated in (C). Left y-axis in lower panel for leukocytes, right y-axis for thrombocytes and hemoglobin. (B) The same blood tests as in (A) at the occasion of the second admission after treatment with nivolumab. Day ‘1’ is the day of treatment, which was also the day of admission in both cases. (C and D) Graphic illustration of the timing of treatment on first (C) and second (D) admission. ALAT, alanine aminotransferase; ASAT, aspartate aminotransferase; CRP, C reactive protein; GGT, gamma-glutamyltransferase, Hb, hemoglobin; Ipi, ipilimumab, Nivo, nivolumabb; PCT, procalcitonin.

One month after diagnosis, the patient was started on palliative first-line therapy with the ICIs ipilimumab (1 mg/kg, total of 64 mg) and nivolumab (3 mg/kg, total of 200 mg) at the Karolinska University Hospital. She received her first course of this combination from 08:30 onwards and left the ambulatory treatment unit without signs of complications. On her way home, she began to feel sick, developed a fever of 39.5°C, and experienced chills and confusion, on which an ambulance was called. She was brought into the emergency department with suspected sepsis. On arrival, her heart rate was 110 /min, her blood pressure was 120/80 mm Hg, and her ECG showed sinus tachycardia without signs of ischemia. Her C-reactive protein (CRP) level on arrival was 102 mg/L (normal: <3 mg/L). In the emergency department, an adverse reaction to the ICI treatment was suspected, and the patient was given antihistamines and 100 mg of hydrocortisone intravenously. Because she continued to have fever and chills, the antibiotic piperacillin/tazobactam was added ex juvantibus. Repeated blood cultures were negative. The patient continuously received both antibiotic and corticosteroid treatment during her inpatient stay, following which decreasing inflammatory parameters were seen (figure 1A, C). The patient was hospitalized for a total of 22 days and was discharged with betamethasone 8 mg per os daily with a planned tapering over a period of almost 2 months. Five weeks after discharge, the patient presented to the outpatient clinic with an improved ECOG performance score of 1. A CT scan showed partial response. A clinical conference decision was made to continue with ICI treatment, but as a monotherapy with nivolumab to reduce the risk of inflammatory side effects. She received the second ICI treatment of 480 mg nivolumab 69 days after the first cycle with ipilimumab and nivolumab. Directly after completion of the infusion, she developed a fever of 38.6°C, sinus tachycardia of 130 beats/min and hypertension of 170/100 mm Hg. Another adverse reaction related to ICI was suspected and she was given hydrocortisone (200 mg intravenously) and paracetamol (1000 mg intravenously); in addition, she was already on treatment with betamethasone 0.5 mg per os daily after the first adverse reaction to ICI. The patient was admitted to the oncological inpatient ward for the second time (figure 1B, D). On admission, the patient’s CRP level was 46 mg/L (normal: <3 mg/L), her procalcitonin level was 5.9 µg/L (normal: >0.5 µg/L), and leukocyte counts were normal. The day after admission, the patient’s vital parameters were stable, serum IL-6 was at 51 ng/L (normal: <7.0 ng/L). Her CRP reached a maximum level of 62 mg/L the day after admission and her procalcitonin level rose to a maximum of 8.2 µg/L 2 days after the ICI infusion. Erythrocyte count, leucocyte count and platelets were all suppressed and reached their nadir 3–4 days post-ICI infusion (figure 1). As the patient had intermittent fever several days after the ICI treatment, piperacillin/tazobactam was administered intravenously for 4 days, and treatment was discontinued when the patient was discharged, as her inflammatory parameters had decreased, and no pathogens were detected in blood cultures.

According to the American Society for Transplantation and Cellular Therapy criteria for grading CRS after CAR T cell therapy,^16^ the patient presented with grades 1–2 CRS on both occasions.

At the first follow-up visit after monotherapy with nivolumab, the patient had elevated transaminases, which was considered as ICI related toxicity, hepatitis grade 3. Because of these severe side effects after monotherapy with nivolumab, ICIs were permanently discontinued. Oral corticosteroids were tapered over several weeks and then discontinued without signs of a recurrent inflammatory flare. Several follow-up CTs showed initially partial response in several metastases and thereafter stable disease. However, a CT scan 10 months following ICI therapy cessation confirmed progressive disease and the patient was switched to a second-line tyrosine kinase inhibitor (TKI). The TKI was discontinued after four months of therapy due to severe treatment-related adverse events, including mucositis, fatigue, and diarrhea. The patient was started on third-line everolimus 18 months after diagnosis and remains free of irAEs.

In summary, this patient developed early-onset, mild CRS that was manageable in a standard oncology ward without the need for vasopressors or invasive ventilation.

### Systematic review

To chart our current knowledge of hyperinflammatory syndromes, such as CRS and HLH, as rare side effects of treatment with ICIs, we conducted a systematic review of the literature housed in the major medical databases as of October 2021. In addition, we manually updated the list of eligible studies as of 1June 13, 2022. Online supplemental figure 2 presents the **P**referred Reporting Items for Systematic Reviews and Meta-Analyses (PRISMA) reporting chart for the study.

10.1136/jitc-2022-005841.supp2 Supplementary data

We identified a total of n=49 articles, reporting on n=189 patients treated in the USA, the UK, Switzerland, Spain, Poland, Lebanon, Japan, Israel, Germany, France, China, Canada, Australia, and Singapore (table 1). Of these studies, n=45 reports were on n=<5 individual patients (comprizing a total of n=56 individuals), while n=4 studies reported case series or data from queries of pharmacovigilance databases. Table 2 summarizes the most important findings from the reports on individual patients. Definitions of CRS/HLH varied significantly between reports, and retrospective diagnostic assessment was not possible due to partially incomplete and heterogeneous clinical data. Therefore, we used the respective authors’ assessment of the hyperinflammatory condition as CRS, HLH, or related hyperinflammatory diseases for further analysis. The earliest published report was from 2016.^13^

**Table 1:** Studies included after systematic review

Authors ref year country	Type and stage	No. of patients	Age* and sex	Antibody	Main complication†	ICI cycle	Time- to-symptoms (days)	Main symptoms	Intervention	CRS/HLH outcome	ICI rechallenge	Tumor- specific outcome
Sharma *et al*^13^ 2016 USA	Lung cancer, ‘progressive’	12	Unclear	Nivolumab	SIRS			‘Fever, tachypnea, tachycardia, hypotension’	Steroids, tocilizumab	Unclear	Unclear	Unclear
El Rassy *et al*.^31^ 2017 Lebanon	Lung squamous cell carcinoma, stage 4	1	60–70 M	Pem brolizumab	CRS	7	1	‘Persistent low-grade fever(…), diffuse swelling’	Furosemide, steroids.	Recovery	Yes, up to at least 13 cycles.	Unclear
Shah *et al*.^46^ 2017 USA	Bladder, stage 4	1	70–80 M	Pem brolizumab	HLH	9 months of therapy	Unclear	Fever, tachycardia, rash, acute renal failure.	Etoposide, steroids	Unclear	Unclear	CR
Rotz *et al*.^47^ 2017 USA	Sarcoma, stage 4	1	20–30 F	Nivolumab	CRS	2	4	Fever, rash, encephalopathy, tachycardia, hypotension.	Steroids, broad-spectrum ABs, tocilizumab.	Recovery	No	Unclear
Urosevic-Maiwald *et al*^48^ 2017 Switzerland	Melanoma, stage 4	2 (1)	40–50 F	Pem brolizumab	SIRS		28	Fever, hypotension, tachycardia, anuria.	ABs, steroids	Recovery	No.	
Urosevic-Maiwald *et al*^48^	Melanoma, stage 4	2 (2)	40–50 F	Pem brolizumab	‘Hhyper sensitivity syndrome’ or DRESS		8	Face swelling, pruritic eruption, hypotension, tachycardia and fever	Steroids	Recovery	No	PD after around 7 months
Takeshita *et al*^49^ 2017 Japan	Squamous lung cancer, stage 4	1	60–70 F	Nivolumab	CRS	2	25	Severe general fatigue and high-grade fever	Steroids	Recovery	No	Regression
Michot *et al*^40^ 2018 France	Melanoma, stage 4	1	50–60 F	Ipilimumab	HLH	1 (8 pembrolizumab)	8 weeks after the last dose of ipilimumab	‘Fever with pancytopenia and disseminated intravascular coagulation’	Steroids, etoposide	Death from brain hemorrhage at metastatic site.	No	Unclear
Sadaat *et al*^50^ 2018 USA	Melanoma, stage 4	1	50–60 M	Pem- brolizumab	HLH	6	31	Fever.	Steroids	Recovery	No	CR for 1 year.
Hantel *et al*^51^ 2018 USA	Melanoma, stage 4	1	30–40 F	Ipilimumab/ nivolumab	HLH	“first doses”	21	Tachycardia, hypotension.	Steroids.	Recovery	No	CR
Satzger *et al*^52^ 2018 Germany	Melanoma, stage 4	1	20–30 F	Ipilimumab/ nivolumab	HLH	4	7	Fever.	Steroids, mycophenolate mofetil.	Recovery	No	CR
Shah and Melissa^53^ 2018 USA	Bladder cancer and thymoma, stage 4	1	70–80 M	Pem brolizumab	HLH			Fever, fatigue				
Sasaki *et al*^54^ 2018 Japan	Melanoma, stage 4	1	60–70 F	Pem brolizumab	HLH	‘1 month after last ICI’	13	Fever, hep atosplenomegaly, erythema multiforme-like lesions.	Steroids	Recovery	No.	PR
Laderian *et al*^55^ 2019 USA	Thymic carcinoma, stage 4	1	40–50 M	Pem brolizumab	HLH	Several over the course of 1 year		Pancytopenia, hemophagocytosis.		Deceased		‘Clinical benefit’
Kogure *et al*^32^ 2019 Japan	Lung adeno- carcinoma, stage 3b	1	60–70 M	Pem brolizumab	CRS	1	2	Fever, tachycardia, hypotension.	Steroids	Recovery	Yes	Pseudoprogression, after 3 cycles of pembrolizumab PR.
Oda *et al*^56^ 2019 Japan	Gastric adeno- carcinoma, stage 4	1	40–50 M	Nivolumab	CRS	1	8	Fever, tachycardia, malaise.	Steroids, mycophenolate mofetil.	Death “from gastric cancer.”	No	PD
Noseda *et al*^18^ 2019 France, Japan, Germany, Switzerland, Canada, USA	diverse	38	M: 29 F: 9	Ipilimumab, nivolumab, pem- brolizumab, atezolizumab, and combinations								
Lorenz *et al*^57^ 2019 Germany	Prostate, stage 4	1	60–70 M	Pem- brolizumab	HLH			Fever, hep atosplenomegaly.	Steroids, plasmapheresis, ciclosporin A, etoposide, tacrolimus.	Recovery	No	CR
Okawa *et al*^58^ 2019 Japan	Lung squamous cell carcinoma, stage 3b	1	70–80 M	Pem- brolizumab	HLH	1	10	Fatigue, fever, jaundice, splenomegaly.	Steroids, ABs.	Recovery	No	CR
Chin *et al*^59^ 2019 Australia	Melanoma, stage 4	1	60–70 F	Nivolumab	HLH		30	Fevers, lethargy, abdominal distention. Hep- atosplenomegaly.	Steroids	Recovery	No	SD
Honjo *et al*^60^ 2019 Japan	Pulmonary pleomorphic carcinoma, stage 3	1	50–60 F	Nivolumab	CRS	‘After the last nivolumab administration’	14	Asthenia, fever. Livedo reticularis with systemic purpura.	Steroids, thrombo modulin and my cophenolate mofetil, hemodi afiltration.	Recovery	No	PR
Adashek and Feldman^61^ 2019 USA	Lung adeno- carcinoma, stage 4	1	70–80 M	Pem- brolizumab	CRS	3 then again after 4	1	Fever, hypotension, mental status change the first time. Then hypotension, respiratory distress.	Tocilizumab	Recovery	Unclear	Unclear
Slota *et al*^20^ 2019 USA	Melanoma, stage 4	1	70–80 M	Nivolumab	CRS	17		Altered mental status, hypotension, tachycardia, fever, hypoxia. Grade 3 maculopapular rash.	ABs, steroids, tocilizumab	Recovery, then relapse 6 weeks later (deceased)	No	PR
Takahashi *et al*^62^ 2020 Japan	Lung adeno- carcinoma, stage 4	1	70–80 M	Pem brolizumab	HLH	1	7	Fever, diarrhea	Steroids, Abs.	Recovery	No	SD for 3 months
Ohira *et al*^63^ 2020 Japan	Renal cell carcinoma, stage 4	1	70–80 M	Nivolumab, ipilimumab	CRS	2	2	"Dermatomyositis (…)high fever, hypotension, respiratory failure, impaired consciousness@	Steroids, mycophenolate mofetil, plasma exchange.	Recovery	No	SD for 2 months
Normand *et al*^64^ 2020 Switzerland	Lung adeno- carcinoma, stage 4	1	70–80 M	Pem brolizumab	CRS	1	1	Fever, renal impairment, confusion, dyspnea.	ABs, steroids.	Recovery	No	SD for 6 months
Gao *et al*^65^ 2020 China	Esophageal squamous cell carcinoma, locally advanced	1	60–70 M	Sintilimab	CRS	3		Fever, diarrhea.	Methyl- prednisolone, tocilizumab, mycophenolate mofetil, immuno globulin.	Recovery	Unclear	Unclear
Özdemir *et al*^66^ 2020 Switzerland	Melanoma, stage 4	3 (1)	40–50 M	Ipilimumab, nivolumab	HLH	2		Fever, nausea, extreme fatigue.	Steroids, tocilizumab, plasma.	Recovery	Unclear	CR
Özdemir *et al*^66^	Melanoma, stage 4	3 (2)	30–40 M	Nivolumab	HLH	5		Splenomegaly, fever and extreme fatigue.	Tocilizumab, steroids, low dose heparin prophylaxis.	Recovery	Unclear	PR
Özdemir *et al*^66^	Melanoma, stage 4	3 (3)	30–40 M	Ipilimumab, nivolumab	HLH	3		Hep atosplenomegaly, fever and fatigue.	Tocilizumab, plasma, low dose heparin prophylaxis	Recovery	Unclear	PR
Azari *et al*^67^ 2020 UK	Renal cell carcinoma, stage 4	1	50–60 M	Nivolumab, ipilimumab	HLH	1	6	‘Myalgia, fevers, frontal headache, photophobia, blurry vision, and vomiting’.	Abs, methyl prednisolone and anakinra.	Recovery	No	Unclear
Dupré *et al*^33^ 2020 France	Pulmonary sarcomatoid carcinoma, stage 4	5 (1)	50–60 M	Pem brolizumab	HLH	1	7	Fever, asthenia, dyspnea.	Steroids, broad-spectrum ABs	Recovery	Yes	PD
Dupré *et al*^33^	Melanoma, stage 4	5 (2)	30–40 F	Ipilimumab, nivolumab	HLH	1	21	Asthenia, splenomegaly	Steroids, etoposide, intravenous immuno globulins, tocilizumab.	Recovery	Yes	SD
Dupré *et al*^33^	Melanoma, stage 4	5 (3)	50–60 F	Ipilimumab, pem brolizumab	HLH	‘After ipilimumab perfusion’	30	Asthenia, fever	Steroids, etoposide	Deceased	No	PD
Cont. Dupré *et al*^33^	Melanoma, stage 4	5 (4)	60–70 M	Ipilimumab, nivolumab	HLH	‘Five weeks and two cycles after the introduction of the combination’.	Unclear	Fever Splenomegaly	Steroids	Recovery	Yes	PD
Dupré *et al*^33^	Melanoma, stage 4	5 (5)	20–30 M	Ipilimumab, nivolumab	HLH			Fever, meningitis, colitis, hepatic cytolysis.	Steroids	Recovery	No	Unclear
Akagi *et al*^68^ 2020 Japan	Lung adeno- carcinoma, stage 3b	1	70–80 M	Pem- brolizumab	HLH	1	27	Joint swelling, hypertension, fever diffuse macular rash.	Steroids, recombinant throm- bomodulin, G-CSF, etoposide.	Recovery	No	CR
Thummalapalli *et al*^69^ 2020 USA	Glioblastoma	1	70–80 M	Nivolumab	HLH	2	17	Fever, altered mental status.	Steroids, Abs.	Deceased	No	Unclear
Mizuta *et al*^70^ 2020 Japan	Melanoma, stage 4	1	60–70 F	Ipilimumab, nivolumab	HLH	2	1	Fever, malaise, headache, grade 2 diarrhea.	NSAID, ABs, steroids	Unclear	Unclear	Unclear
Ceschi *et al*^19^ 2020 WHO database, inc. reports from N. America, Europe, Australia, Japan.	Melanoma, lung cancer, others	58 cases, 42 patients with non-hemato logical malignancies.	Med. age 55 y (incl. hemato logical cases). M:34 F: 21	Ipilimumab, nivolumab, pem brolizumab, cemiplimab, atezolizumab, avelumab a nd combinations.	CRS	10 cases after a single administration.	‘A median of 4 weeks’	‘CRS, defined accordingly to the correspondent MedDRA PT ‘cytokine release syndrome’ (MedDRA version 21.1)’.	No reported	Two fatal cases, unknown outcome in 20 cases	Unclear	Unclear
Kalmuk *et al*^34^ 2020 USA	Oropharyngeal squamous cell carcinoma, stage 4	1	60–70 M	Pem- brolizumab	HLH	14	4	Fever, malaise.	Antibiotics, steroids, etoposide	Recovery	Yes	SD for 8 months
Hu *et al*^71^ 2020 China	Colon cancer, stage 4	1	50–60 M	Sintilimab	cytokine storm	2	1	Fever, hypotension, dyspnea.	IV fluids, vasopressors, steroids, ABs, nintedanib.	Recovery	Unclear	Unclear
Nieves-Borrero *et al*^72^ 2020 USA	Small cell lung cancer, metastatic	1	60–70 M	Atezolizumab	CRS	1	3	Hypotension, cardiac arrest.	Hemodialysis, renal replacement therapy.	Deceased	No	Unclear
Dudda *et al*^73^ 2020 Germany	Melanoma, stage 4	1	Unclear Unclear	Nivolumab	HLH		21	Splenomegaly, fever	Broad-spectrum Abs.	Recovery	No	PR
Amlani *et al*^74^ 2020 Canada	Melanoma, stage 4	1	50–60 M	Nivolumab	CRS			Fatigue, nausea, vomiting, diarrhea, fever, and a purpuric eruption. Shock.	Broad-spectrum ABs, steroids, tocilizumab	Recovery	No	Unclear
Yomota *et al*^75^ 2021 Japan	Lung adeno- carcinoma, stage 4	1	50–60 M	Atezolizumab	CRS	1	7	‘High fever, rash, DIC, reduced level of consciousness, heart failure…’.	Steroids, tocilizumab from day 11.	Recovery	No	PR
Percik *et al*.^76^ 2021 Israel	Melanoma, stage 3b	2 (1)	50–60 M	Nivolumab, ipilimumab	Capillary-leak syndrome		21	Generalized edema	Discontinuation of ICI	Initial recovery; proximal muscle weakness and death 1 month later.	No	PR
Percik *et al*^76^	Duodenal adeno- carcinoma, stage 2	2 (2)	70–80 F	Pem brolizumab and ‘an investigational CTLA-4 blocker.’	Capillary-leak syndrome		1	Fever, fatigue, bilateral leg swelling, weight gain.	Steroids	Recovery	Unclear	Unclear
Del Bello *et al*^77^ 2021 France	Squamous cell carcinoma, unclear stage	1	80–90 M	Cemiplimab	Cytokine storm.	1	7	‘Septic shock’.	Steroids	Dialysis, kidney necrosis trans- plantectomy. complications after surgery leading to death.	No	Unclear
Sackstein *et al*^78^ 2021 USA	Lung adeno carcinoma, stage 4	1	50–60 M	Pem brolizumab	CRS	3	19	Fever, chills, hypotension, tachypnea, lethargy.	ABs, steroids, ivermectin, hemodialysis, tocilizumab	Recovery	Unclear	Unclear
Olivares-Hernández *et al*^79^ 2021 Spain	Choroidal melanoma, stage 4	1	70–80 F	Ipilimumab	HLH	3		Fever, splenomegaly	Steroids, tocilizumab.	Recovery	Unclear	PR
Kurozumi *et al*^80^ 2021 Japan	Lung adeno- carcinoma, stage 4	2 (1)	70–80 M	Pem brolizumab	HLH	1	10	Fever	Steroids	Unclear	Unclear	Unclear
Kurozumi *et al*^80^	Lung adeno carcinoma. Stage 3b	2 (2)	60–70 F	Pem brolizumab	HLH	‘After last dose of pembrolizumab’.	30	Cytopenia, elevated liver enzyme levels.	Steroids	Unclear	Unclear	Unclear
Masood *et al*^81^ 2021 USA	Renal cell carcinoma, stage 4	1	60–70 M	Ipilimumab, nivolumab	HLH			Generalized weakness. intermittent fevers, splenomegaly.	Steroids	Recovery	Unclear	Unclear
Pacholczak-Madej *et al*^82^ 2021 Poland	Melanoma, stage 4	1	50–60 F	Ipilimumab, nivolumab	HLH	4		Fever, general malaise, dyspnea, splenomegaly.	Steroids, FFP, mycophenolate mofetil, cyclophos- phamide, etoposide, ciclosporin.	Recovery	No	PR
Tiu *et al*^83^ 2021 UK	Lung carcinoma, stage 4	3 (1)	50–60 F	Unclear	HLH	1	11	Fever, rigors.	Broad-spectrum ABs, steroids.	Recovery	Unclear	Unclear
Tiu *et al*^83^	Breast cancer, stage 4	3 (2)	40–50 F	Unclear	HLH	1	11	Fever, maculopapular rash, dyspnea, hypoxia.	ABs, steroids, tocilizumab.	Recovery	Unclear	Unclear
Tiu *et al*^83^	Bladder cancer, stage 4	3 (3)	60–70 M	Unclear	HLH	1	10	Fever, rigors.	ABs, steroids, tocilizumab, siltuximab, anakinra, plasma exchange, intravenous immunoglobulins.	Recovery	Unclear	Unclear
Tay *et al*^17^ 2022 Singapore	NSCLC, renal cell carcinoma, hepatocellular carcinoma, melanoma	25	Med. age 64 M: 18 F: 7	Pem brolizumab, nivolumab, ipilimumab, anti-LAG3-antibody.	CRS		Median of 11 days	All had fever of 38°C or higher.	Steroids, tocilizumab	three fatal	In 7 patients, no grade 3/4 events.	PR: 6, SD: 5, P D: 10
Zhang *et al*^84^ 2022 China	Lung adeno carcinoma, stage 4	1	60–70 F	Pem brolizumab	CRS		1	Fever, nausea, vomiting, chest pain.	Broad-spectrum ABs, intravenous fluids, steroids.	Recovery	No	PR

*Age given in ranges due to journal constraints.

†As assessed by the authors of the respective study.

AB, antibiotics; CR, complete response; CRS, cytokine-release syndrome; EBV, Epstein-Barr virus; F, female; HLH, hemophagocytic lymphohistiocytosis; ICI, immune checkpoint inhibitor; M, male; PR, partial response; SD, stable disease; SIRS, systemic inflammatory response syndrome.

**Table 2:** Summary of the studies reporting on individual patients

Tumor-specific outcome (per individual patient)	
Complete response	8 (14%)
Partial response	13 (23%)
Stable disease	6 (11%)
Progressive disease	4 (7%)
Unspecified	25 (45%)
Type of hyperinflammatory syndrome	
CRS	16 (28%)
HLH	34 (61%)
other	6 (11%)
Type of ICI	
Pembrolizumab	21 (38%)
Nivolumab	11 (20%)
Ipilimumab	2 (4%)
Atezolizumab	2 (4%)
Ipilimumab+nivolumab	13 (23%)
Cemiplimab	1 (2%)
Ipilimumab+pembrolizumab	1 (2%)
Sintilimab	2 (4%)
Unspecified	3 (5%)
CRS/HLH treatment with IL-6 blockade	
Yes	14 (25%)
No	42 (75%)
Rechallenge with ICIs	
Yes	6 (11%)
No	31 (55%)
Unspecified	19 (34%)
CRS/HLH outcome	
Recovery	43 (77%)
Deceased, related to CRS/HLH	6 (11%)
Deceased, other reasons	2 (4%)
Unspecified	5 (9%)
Time to CRS/HLH onset	
Days after last administration*	9 (3,75-21)
Cycles before symptoms onset*	2(1-3)
Type of underlying malignancy	
Bladder cancer	3 (5%)
Lung cancer	15 (27%)
Malignant melanoma	21 (38%)
Renal cell carcinoma	3 (5%)
Other	14 (25%)

*Numbers given as mean with IQR

CRS, cytokine release syndrome; HLH, hemophagocytic lymphohistiocytosis; ICI, immune-checkpoint inhibitor.

The ICIs used included pembrolizumab (n=21 studies) and nivolumab (n=11 studies) as single agents, combined treatment with ipilimumab and nivolumab (n=13), and less frequently (n=2 studies each), ipilimumab, sintilimab or atezolizumab monotherapy, and cemiplimab (n=1). Often, ICIs were combined either with chemotherapeutical agents or other anticancer drugs, such as TKIs (ie, cabozantinib or dabrafenib/trametinib), or drugs that were used prior to development of the hyperinflammatory syndrome(s).

In the individual reports, n=16 patients were diagnosed with CRS, n=34 with HLH, and n=6 were described as having related hyperinflammatory conditions such as capillary leak syndrome with high fever, or systemic inflammatory response syndrome. The most frequently reported underlying malignancies in individual case reports were malignant melanoma (n=21 individual reports) and lung cancer (n=15 individual reports). The average age of the patients in the individual reports was 59 years. In individual reports that included the patients’ sex, n=35 patients were males, and n=20 patients were females. Higher frequencies of male patients were also reported in the largest case series from two centers in Singapore^17^ (n=18 males, n=7 females), and in a WHO pharmacovigilance database queried for HLH^18^ (n=29 males, n=9 females) and CRS, respectively^19^ (n=34 males, n=21 females). Most studies (n=46 studies out of 49) comprised data on patients with metastatic disease (table 1). Time from ICI infusion to onset of symptoms varied from hours to 1 month, with a median of n=9 days. The number of cycles of ICIs received prior to CRS diagnosis varied between n=1 and n=17, with a majority of cases developing CRS after more than one ICI administration (n=16 patients after one cycle of ICI, n=31 patients after more one than one cycle of ICI). In total, n=42 studies on n=56 individual patients (75%) reported recovery from ICI-induced irAEs, while n=6 cases (out of 56 [11%]) were fatal. Of those, n=3 cases were reported to have HLH, and n=3 had CRS, as per the authors’ assessment. Overall, n=42 patients from individual reports were reported to have recovered from their hyperinflammatory complications. Reports from pharmacovigilance data were incomplete for outcome data; a study including n=25 patients from Singapore reported n=3 fatal cases (0.12 %).

Treatments for CRS/HLH varied significantly and included different types of corticosteroids in almost all cases, and tocilizumab in n=14 patients (25% of all individual cases). None of the patients treated with tocilizumab had a fatal outcome after the reported CRS/HLH episode but one patient relapsed with CRS six weeks after the first episode and passed away despite tocilizumab treatment, although no further information on the second episode was provided.^20^ In contrast, fatal outcome was reported for n=6 patients in the remaining n=42 individual patients where tocilizumab was not mentioned as treatment (corresponding to 14%). Various other drugs were used in some patients, including etoposide in combination with dexamethasone, an established HLH treatment,^21 22^ as well as intravenous immunoglobulins, plasmapheresis, mycophenolate mofetil, and tacrolimus. Often, these were combined, at least initially, with antibiotic therapy because of suspected sepsis (table 1).

## Discussion

Wider indications have continuously led to higher numbers of patients being treated with ICIs.^23^ Hence, the clinical need to understand rare side effects has increased, and higher patient numbers allow more informed clinical decisions. CRS and HLH are potentially life-threatening side effects of treatment with ICIs; however, they are relatively rare and therefore are challenging to study. In this systematic review, we provide a comprehensive picture of the reported cases in the literature to date. Our results underscore the notion that hyperinflammatory syndromes are rare, often treatable, and that fatal outcome occurs in a minority of the cases (table 2). It is interesting to note the predominance of male patients in case reports and case series, as well as in pharmacovigilance databases. This might partly be driven by a higher proportion of men among patients with lung cancer,^24^ which represent the second largest tumor group in the identified studies, and partly by sex differences in the immune response.^25^ Interestingly, pharmacovigilance data suggest that the outcome of CRS is more favorable in females than in males,^19^ warranting further research into sex differences in response to treatment and in side effect profiles of ICIs.

A diagnostic challenge that occurs with rapid onset of fever following ICI administration is to differentiate between infusion-related reactions and CRS/HLH. In the case presented here, the symptoms developed early after the start of treatment; the persistence of the hyperinflammatory state for days, together with elevated IL-6 suggest CRS rather than a hypersensitivity reaction as the cause, although there is considerable overlap in the symptoms and the cellular mechanisms of CRS and hypersensitivity reactions.^26^

As illustrated by our case report, a common clinical dilemma in patients receiving ICIs is whether to continue treatment despite severe irAEs. Key factors in this decision-making process are the risk of recurrence of a given irAE, the anticipated severity in case of recurrence, as well as its treatability. A recent study has suggested that IL-6 blockade given in parallel with ICIs ameliorates irAEs, while enhancing the antitumoral effect of ICIs.^27^ Earlier experimental data had already hinted at the potential benefit of adding IL-6 inhibitors to ICIs in mouse models of pancreatic cancer^28^ (which is largely resistant to ICI therapy), as well as hepatocellular carcinoma.^29^ Together, these data suggest that IL-6 blockade does not abrogate, but rather enhances, the activation of a beneficial antitumoral immune response, providing a potential oncological rationale for combining IL-6 inhibition and ICIs. Results from CAR-T cell therapy in hematological malignancies support the lack of antagonistic effects of IL-6 antagonists in combination with ICIs; for example, in refractory large B-cell lymphoma, response rates to CAR T cell therapy were independent of the use of concomitant tocilizumab to treat CRS.^30^

Our review of the literature revealed no fatal case among 14 patients who received the IL-6 inhibitor, tocilizumab, for CRS/HLH treatment, while the fatality rate was 14% in the patients for which anti-IL-6 treatment was not reported. One patient who had received tocilizumab passed away after a second CRS episode, but further details on the suspected CRS trigger and clinical course were not provided.^20^ Since the groups of patients are gathered from case reports and hence are not comparable, we cannot conclude that IL-6 inhibition is beneficial. Nevertheless, given the evidence that combined anti-IL-6/ICI treatment enhances the antitumoral effect, an important question is whether a rechallenge after mild CRS/HLH that responded well to anti-IL-6 treatment or corticosteroids should be considered. As summarized in table 2, only a few case reports exist on ICI rechallenge after CRS or HLH, none of which reports fatal outcomes after rechallenge.^31–34^ In the largest case series published containing n=25 cases, n=7 were cases after rechallenge with ICIs; none of these had grade 3 or 4 CRS,^17^ which lends some support for continuous ICI treatment despite CRS and could be appropriate in selected cases, as the risk of aggravated side effects when re-exposing patients to ICIs could be low. Furthermore, a meta-analysis of patients with non-small cell lung cancer rechallenged with ICIs suggests that certain patients with disease progression during ICI discontinuation might benefit from ICI rechallenge.^35^ Whether these data are applicable to ICI-related CRS remains to be seen.

In the case of the patient presented in the current report, the second occasion of CRS was milder than the first, although a switch from combined ipilimumab/nivolumab to nivolumab alone might have contributed to the milder clinical course on the second occasion. In addition, the patient was treated with oral betamethasone on rechallenge with nivolumab, which could have prevented severe CRS, although some authors have contested the preventive effect of steroids, at least when using bispecific antibodies.^36^ As a higher tumor burden has been shown to correlate with increased inflammation parameters and cytokine levels,^37^ it can be hypothesized that pretreatment tumor burden is associated with the risk of developing irAEs. Indeed, studies of CAR T cell treatment for B cell acute lymphoblastic leukemia have shown a significant correlation between severe CRS and a high tumor burden, which might lead to amelioration of CRS in case of treatment effects of previous courses^38 39^.

While we mostly found case reports, two studies reported pharmacovigilance data from the global WHO database, and two reports queried the Registry of Severe Adverse Reactions to Immunomodulatory Antibodies used in Oncology (REISAMIC), a database for ICI-related irAEs in France.^33 40^ The first report on REISAMIC data included n=16 patients with ‘fever reaction’ to ICI treatment, and based on their analysis of these cases, the authors concluded that this irAE can ‘usually be controlled with a short course of corticosteroids’. The second report also queried two other French databases specifically for HLH cases, and identified n=5 patients with HLH, one of which was fatal. In n=3 of the five cases, rechallenge of ICI was reported, in n=2 cases without recurrence of fever of HLH.^33^ One of the HLH cases was identified in REISAMIC among n=745 patients included ‘at a single center between 2014 and 2019’, suggesting that HLH indeed is a rare event. The rarity of HLH is also supported by the analysis of WHO pharmacovigilance data: 49,883 ICI-related adverse events were retrieved from the WHO database VigiBase on a search conducted in September 2018, n=38 of whom corresponded to HLH, and n=34 were directly linked to ICI treatment, usually developing more than 6 weeks after ICI treatment. Interestingly, the rate of other irAEs was below 20%.^18^ The same group of authors queried VigiBase for CRS as of January 2020. They found n=58 reports likely corresponding to CRS among a total of 80 700 reports on ICI-related adverse events, of which n=43 were definitely related to ICIs, and which occurred a median of approximately 4 weeks after initiation of ICI treatment.^19^ Two of those cases were fatal. Finally, a recent study presented a case series collected at two hospitals in Singapore between February 2014 and January 2021. They found that n=25 out of a total of n=539 patients that had received ICI developed CRS, which is a considerably higher frequency than suggested by the pharmacovigilance data, and the reason for this difference is unclear. In this cohort, n=7 patients with low-grade CRS were rechallenged with ICIs and did not relapse. A total of n=3 cases had fatal CRS despite tocilizumab treatment.^17^ The authors also suggested that time-to-fever-onset, low platelet count, and high urea levels at CRS presentation might serve as indicators of a severe course.^17^

Several studies have reported an association between irAEs and improved treatment outcome,^41 42^ although the type of irAE might predict treatment outcome, since specific irAEs, such as pneumonitits, might not predict ICI efficacy (reviewed in ref.^41^). We find that n=27 individual patients with hyperinflammatory syndromes had clinical benefit (CR in n=8 patients, PR in n=13 patients, SD in n=6 patients) from ICI treatment, while only n=4 patients had PD. Although follow-up time frames and assessment methods will vary substantially between studies and were inconsistently reported, it is tempting to speculate that CRS, like other irAEs, might be associated with improved treatment outcomes. A potential pitfall in this interpretation is that many of the studies assessed did not provide information about patient treatment outcome, potentially providing a bias toward the patients with beneficial treatment outcome.

It is still unclear whether ICI rechallenge after irAEs might be oncologically favorable. In accordance with current guidelines, most patients with grade 3 or 4 irAEs will discontinue treatment with ICI permanently. The rarity of cases, resulting in cohorts of limited size, poses a challenge when addressing this question. While recent studies have shown that rechallenge with ICI after irAE does not significantly improve overall survival,^43^ others have concluded that irAEs on rechallenge display milder toxicities, and suggested that rechallenge might be safe for most patients, depending on the type of irAE.^44 45^

In summary, the published data suggest that CRS and HLH are infrequent, potentially severe, but frequently treatable side effects of ICIs, and that rechallenge could be considered in selected cases, with IL-6 inhibition as an attractive preventive and therapeutic option.
