# Supplementary material for: Systemic inflammatory syndromes as life-threatening side effects of immune checkpoint inhibitors: case report and systematic review of the literature
Source: J Immunother Cancer. 2023 Mar 6;11(3):e005841. doi: 10.1136/jitc-2022-005841 (PMC9990684; doi:10.1136/jitc-2022-005841)
Supplement: Supplementary data [file jitc-2022-005841supp001.pdf]

Supplementary Figure 1: Search strategy and search terms

1. Medline

Interface: Ovid MEDLINE(R) and Epub Ahead of Print, In-Process & Other Non-Indexed Citations and Daily<br><br>Date of Search: 26th October 2021<br><br>Number of hits: 444<br><br>Comment: In Ovid, two or more words are automatically searched as phrases; i.e. no quotation marks are needed	Field labels<br><br>• exp/ = exploded MeSH term<br>• / = non exploded MeSH term<br>• .ti,ab,kf. = title, abstract and author keywords<br>• adjx = within x words, regardless of order<br>• * = truncation of word for alternate endings

Database(s): **Ovid MEDLINE(R) and Epub Ahead of Print, In-Process, In-Data-Review & Other Non-Indexed Citations and Daily** 1946 to October 25, 2021
Search Strategy:

#	Searches	Results
1	Cytokine Release Syndrome/	1316
2	Interleukin-1/	35705
3	Interleukin-6/	67997
4	Systemic Inflammatory Response Syndrome/	6384
5	(cytokine* adj3 (storm* or syndrome*)).ti,ab,kf.	6121
6	((inflammatory response or multi-system inflammatory or sepsis or septic or systemic inflammat*) adj3 syndrome*).ti,ab,kf.	9329
7	(hypercytokinemia* or hypercytokinaemia* or interleukin 1 or interleukin1 or interleukin i or il-1 or il1 or interferon beta 2 or interferon beta2 or interleukin b or interleukin hp1 or interleukin 6 or interleukin6 or IL6 or IL 6).ti,ab,kf.	199037
8	((b-cell differentiation or b-cell stimulatory or b lymphocyte stimulating or hepatocyte stimulating or hybridoma or plasmacytoma) adj2 factor*).ti,ab,kf.	710
9	or/1-8	231624
10	Macrophage Activation Syndrome/	557
11	Lymphohistiocytosis, Hemophagocytic/	3250
12	(macrophage activati* adj2 syndrome*).ti,ab,kf.	1234
13	((Erythrophagocyt* or hemophagocyt*) adj2 (histiocyt* or hymphohistiocyt* or lymphohistiocyt* or reticulosis or syndrome*)).ti,ab,kf.	5341
14	or/10-13	7028

Supplementary Figure 1: Search strategy and search terms

Karolinska Institutet

15	9 or 14	237794
16	Immune Checkpoint Inhibitors/	2924
17	Ipilimumab/	2300
18	Nivolumab/	3587
19	(atezolizumab or avelumab or cemiplimab or durvalumab or pembrolizumab).nm.	3755
20	(atezolizumab or avelumab or cemiplimab or durvalumab or ipilimumab or nivolumab or pembrolizumab).ti,ab,kf.	13171
21	((CTLA-4 or CTLA4 or Cytotoxic T-Lymphocyte-Associated Protein 4 or checkpoint or PD-1 or PD1 or PD-L1 or PDL1 or Programmed Death-Ligand 1 or Programmed Cell Death Protein 1) adj3 (block* or inhibit*)).ti,ab,kf.	30253
22	(Anti-CTLA-4 MAb or strentarga or yervoy).ti,ab,kf.	169
23	(anti-PDL1 or anti-PD L1 or monoclonal antibody mpdl 3280a or monoclonal antibody mpdl3280a or tecentriq).ti,ab,kf.	2132
24	(bavencio or imfinzi or keytruda or lambrolizumab or libtayo).ti,ab,kf.	135
25	or/16-24	38419
26	15 and 25	527
27	26 not (animals not humans).sh.	465
28	limit 27 to yr="2013 -Current"	444

Supplementary Figure 1: Search strategy and search terms

Karolinska Institutet

2. Embase

Interface: embase.com<br><br>Date of Search: 26th October 2021<br><br>Number of hits: 1,579<br><br>Comment: Emtree is the controlled vocabulary in Embase	Field labels<br><br>• /exp = exploded Emtree term<br>• /de = non exploded Emtree term<br>• ti,ab,kw = title, abstract and author keywords<br>• NEAR/x = within x words, regardless of order<br>• * = truncation of word for alternate endings

No.	Query	Results
#1	'cytokine storm'/exp	10555
#2	'interleukin 1'/de	67261
#3	'interleukin 6'/de	279870
#4	'systemic inflammatory response syndrome'/de	13389
#5	(cytokine* NEAR/3 (storm* OR syndrome*)):ti,ab,kw	9621
#6	(('inflammatory response' OR 'multi-system inflammatory' OR sepsis OR septic OR 'systemic inflammat*') NEAR/3 syndrome*):ti,ab,kw	14236
#7	hypercytokinemia*:ti,ab,kw OR hypercytokinaemia*:ti,ab,kw OR 'interleukin 1':ti,ab,kw OR interleukin1:ti,ab,kw OR 'interleukin i':ti,ab,kw OR 'il-1':ti,ab,kw OR il1:ti,ab,kw OR 'interferon beta 2':ti,ab,kw OR 'interferon beta2':ti,ab,kw OR 'interleukin b':ti,ab,kw OR 'interleukin hp1':ti,ab,kw OR 'interleukin 6':ti,ab,kw OR interleukin6:ti,ab,kw OR il6:ti,ab,kw OR 'il 6':ti,ab,kw	274664
#8	(('b-cell differentiation' OR 'b-cell stimulatory' OR 'b lymphocyte stimulating' OR 'hepatocyte stimulating' OR hybridoma OR plasmacytoma) NEAR/2 factor*):ti,ab,kw	787
#9	#1 OR #2 OR #3 OR #4 OR #5 OR #6 OR #7 OR #8	401096
#10	'hemophagocytic syndrome'/exp	11879
#11	('macrophage activati*' NEAR/2 syndrome*):ti,ab,kw	2383
#12	((erythrophagocyt* OR hemophagocyt*) NEAR/2 (histiocyt* OR hymphohistiocyt* OR lymphohistiocyt* OR reticulosis OR syndrome*)):ti,ab,kw	8173
#13	#10 OR #11 OR #12	13379
#14	#9 OR #13	412207
#15	'immune checkpoint inhibitor'/de	8085
#16	'avelumab'/de	3790
#17	'atezolizumab'/de	8825
#18	'cemiplimab'/de	724
#19	'durvalumab'/de	6008

Supplementary Figure 1: Search strategy and search terms

Karolinska Institutet

#20	'ipilimumab'/de	17977
#21	'nivolumab'/de	25266
#22	'pembrolizumab'/de	23295
#23	atezolizumab:ti,ab,kw OR avelumab:ti,ab,kw OR cemiplimab:ti,ab,kw OR durvalumab:ti,ab,kw OR ipilimumab:ti,ab,kw OR nivolumab:ti,ab,kw OR pembrolizumab:ti,ab,kw	30924
#24	(('ctla-4' OR ctla4 OR 'cytotoxic t-lymphocyte-associated protein 4' OR checkpoint OR 'pd-1' OR pd1 OR 'pd-l1' OR pdl1 OR 'programmed death-ligand 1' OR 'programmed cell death protein 1') NEAR/3 (block* OR inhibit*)):ti,ab,kw	55830
#25	'anti-ctla-4 mab':ti,ab,kw OR strentarga:ti,ab,kw OR yervoy:ti,ab,kw	316
#26	'anti-pdl1':ti,ab,kw OR 'anti-pd l1':ti,ab,kw OR 'monoclonal antibody mpdl 3280a':ti,ab,kw OR 'monoclonal antibody mpdl3280a':ti,ab,kw OR tecentriq:ti,ab,kw	5479
#27	bavencio:ti,ab,kw OR imfinzi:ti,ab,kw OR keytruda:ti,ab,kw OR lambrolizumab:ti,ab,kw OR libtayo:ti,ab,kw	319
#28	#15 OR #16 OR #17 OR #18 OR #19 OR #20 OR #21 OR #22 OR #23 OR #24 OR #25 OR #26 OR #27	85919
#29	#14 AND #28	2602
#30	#29 NOT ([animals]/lim NOT [humans]/lim)	2277
#31	#30 AND ('Conference Abstract'/it OR 'Conference Paper'/it)	631
#32	#30 NOT #31	1646
#33	#32 AND (2013:py OR 2014:py OR 2015:py OR 2016:py OR 2017:py OR 2018:py OR 2019:py OR 2020:py OR 2021:py OR 2022:py)	1579

Supplementary Figure 1: Search strategy and search terms

Karolinska Institutet

3. Web of Science Core Collection

Interface: Clarivate Analytics<br><br>Date of Search: 26th October 2021<br><br>Number of hits: 632	Field labels<br><br>• TS/Topic = title, abstract, author keywords and Keywords Plus<br>• NEAR/x = within x words, regardless of order<br>• * = truncation of word for alternate endings<br><br>Note: sometimes “quotation marks” are needed for single search terms to avoid automatic term mapping (lemmatization).

#1	TS=(cytokine* NEAR/2 (storm* or syndrome*))	6,944
#2	TS=(("inflammatory response" OR "multi-system inflammatory" OR "sepsis" OR "septic" OR "systemic inflammat*") NEAR/2 syndrome*)	10,737
#3	TS=(hypercytokinemia* OR hypercytokinaemia* OR "interleukin 1" OR "interleukin1" OR "interleukin i" OR "il-1" OR "il1" OR "interferon beta 2" OR "interferon beta2" OR "interleukin b" OR "interleukin hp1" OR "interleukin 6" OR "interleukin6" OR "IL6" OR "IL 6")	278,354
#4	TS=(("b-cell differentiation" OR "b-cell stimulatory" OR "b lymphocyte stimulating" OR "hepatocyte stimulating" OR "hybridoma" OR "plasmacytoma") NEAR/1 factor*)	875
#5	TS=("macrophage activati*" NEAR/1 syndrome*)	2,067
#6	TS=((erythrophagocyt* OR hemophagocyt*) NEAR/1 (histiocyt* OR hymphohistiocyt* OR lymphohistiocyt* OR "reticulosis" OR syndrome*))	7,347
#7	#6 OR #5 OR #4 OR #3 OR #2 OR #1	300,364
#8	TS=(atezolizumab OR avelumab OR cemiplimab OR durvalumab OR ipilimumab OR nivolumab OR pembrolizumab)	28,861
#9	TS=(("CTLA-4" OR "CTLA4" OR "cytotoxic t-lymphocyte-associated protein 4" OR "checkpoint" OR "PD-1" OR "PD1" OR "PD-L1" OR "PDL1 "OR "programmed death-ligand 1" OR "programmed cell death protein 1") NEAR/2 (block* OR inhibit*))	37,463
#10	TS=("Anti-CTLA-4 MAb" OR "strentarga" OR "yervoy")	159
#11	TS=("anti-PDL1" OR "anti-PD L1" OR "monoclonal antibody mpdl 3280a" OR "monoclonal antibody mpdl3280a" OR "tecentriq")	3,108
#12	TS=("bavencio" OR "imfinzi" OR "keytruda" OR "lambrolizumab" OR "libtayo")	150
#13	#12 OR #11 OR #10 OR #9 OR #8	55,381
#14	Refined by: PUBLICATION YEARS: (2021 OR 2015 OR 2020 OR 2014 OR 2019 OR 2013 OR 2018 OR 2017 OR 2016) Indexes=SCI-EXPANDED, SSCI, A&HCI, ESCI Timespan=1945-2021	670

Supplementary Figure 1: Search strategy and search terms

Karolinska Institutet

#15	#13 AND #7<br>Refined by: PUBLICATION YEARS: (2021 OR 2015 OR 2020 OR 2014 OR 2019 OR 2013 OR 2018 OR 2017 OR 2016)<br>*Indexes=SCI-EXPANDED, SSCI, A&HCI, ESCI Timespan=1945-2021*	632
