# Supplementary figures and images for: Systemic inflammatory syndromes as life-threatening side effects of immune checkpoint inhibitors: case report and systematic review of the literature
Source: J Immunother Cancer. 2023 Mar 6;11(3):e005841. doi: 10.1136/jitc-2022-005841 (PMC9990684; doi:10.1136/jitc-2022-005841)

Supplementary Figure 2: PRISMA 2020 diagram

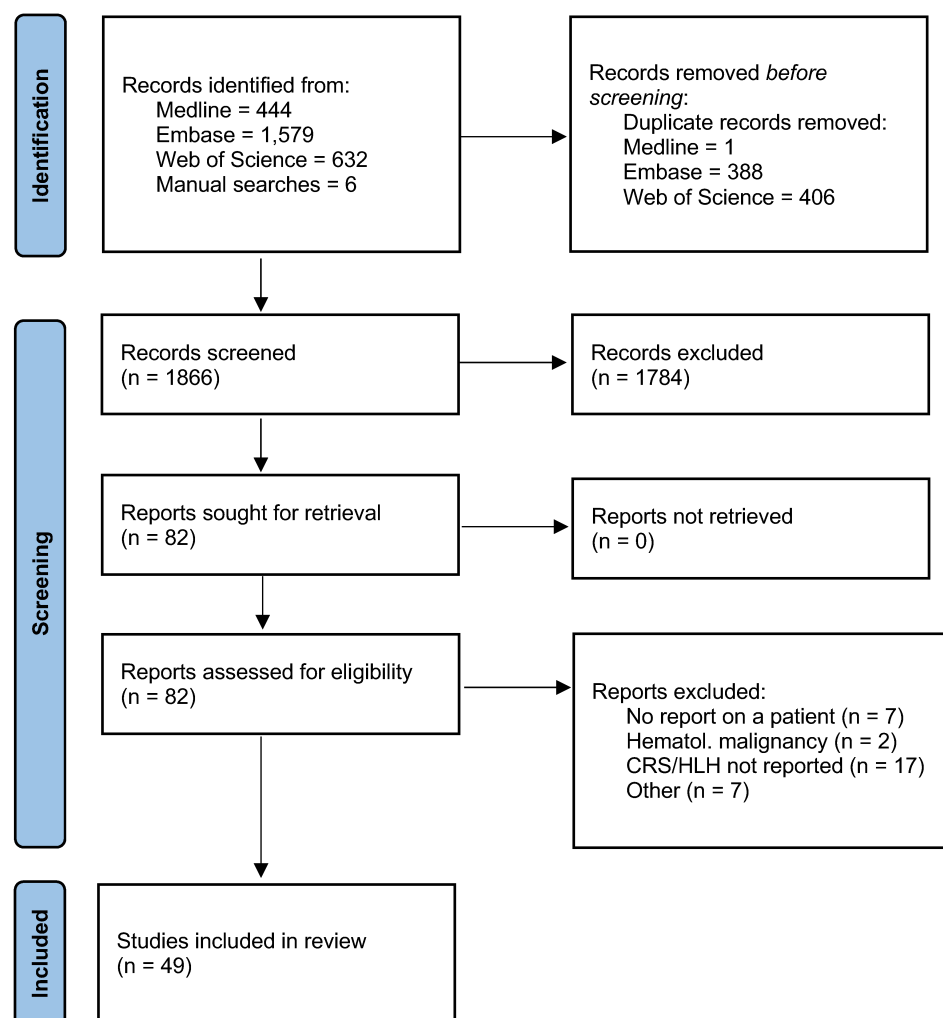

Supplement: Supplementary data [file jitc-2022-005841supp002.pdf]
